# Capsaicin Mitigates Insulin Resistance‐Driven Prostate Hyperplasia Via IGF‐1 Signalling Modulation in HFD + STZ Rat Model

**DOI:** 10.1002/jbt.70845

**Published:** 2026-04-16

**Authors:** Manvitha Kotaru, Nihar Ranjan Das

**Affiliations:** ^1^ Department of Pharmacology, GITAM School of Pharmacy GITAM Deemed to be University Visakhapatnam India

**Keywords:** high‐fat diet, hyperinsulinemia, IGF‐1, insulin resistance, prostatic hyperplasia

## Abstract

Insulin Resistance (IR) is usually associated with elevated glucose levels and hyperinsulinemia. Elevated insulin levels during IR contribute to prostate enlargement and can lead to the condition of Benign Prostatic Hyperplasia (BPH). This study investigated the effects of capsaicin, a bioactive compound derived from chilli peppers, on IR‐induced prostatic hyperplasia. High‐fat diet (HFD) combined with streptozotocin (STZ) was used to induce IR in rats. Rats subjected to HFD + STZ exhibited elevated fasting blood glucose, insulin levels and an increased Prostate weight, confirming IR‐induced prostate enlargement. Capsaicin administration significantly ameliorated fasting glucose and insulin levels, reduced prostate weight and mitigated histopathological alterations. Furthermore, capsaicin treatment downregulated IGF‐1 expression, suggesting its potential role in modulating IGF‐1 signalling involved in cellular proliferation and differentiation and reversing prostate hyperplasia. These findings open up new avenue for exploring more about capsaicin as a possible therapeutic candidate for managing IR‐induced prostatic hyperplasia.

## Graphical abstract

High‐fat diet with streptozotocin induced Insulin Resistance (IR) with elevated fasting blood glucose, insulin levels and increased Prostate weight. Capsaicin administration significantly ameliorated fasting glucose, insulin levels, reduced prostate weight and downregulated IGF‐1, MEK and ERK expression, and mitigated histopathological alterations suggesting its potential role in cellular proliferation and differentiation and reversing prostate hyperplasia.

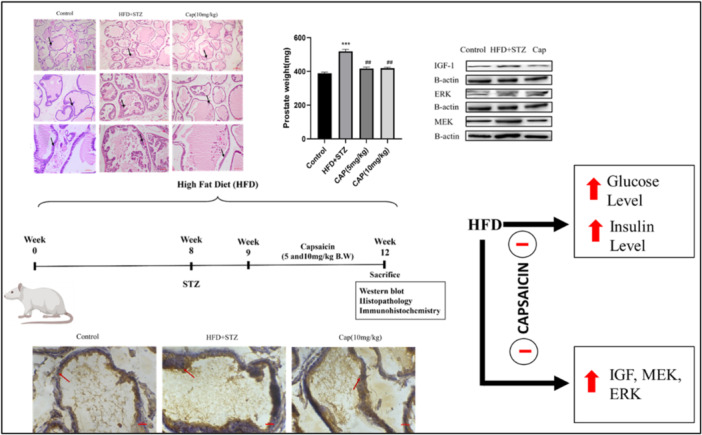

**Abbreviations**
AUC: Area under curve
BPH: Benign prostatic hyperplasia
Cap: Capsaicin
DAB: 3, 3’‐diaminobenzidine
ELISA: Enzyme‐linked immunosorbent assay
ERK: Extracellular signal regulated kinase
HFD: High‐fat diet
H&E: Haematoxylin and eosin
IGF‐1: Insulin‐like growth factor‐1
IGF‐1R: Insulin‐like growth factor receptor‐1
IHC: Immunohistochemistry
IR: Insulin Resistance
MEK: Mitogen‐activated protein kinase
OGTT: Oral glucose tolerance test
PVDF: Polyvinylidene fluoride
RIPA: Radioimmunoprecipitation Assay
SDS‐page: Sodium dodecyl‐sulfate polyacrylamide gel electrophoresis
STZ: Streptozotocin

## 1 Introduction

During Insulin Resistance (IR), to compensate the elevated glucose levels, insulin is secreted more causing a condition called hyperinsulinemia which may lead to Benign Prostatic Hyperplasia (BPH) [1]. IR with high fasting blood glucose and hyperinsulinemia is commonly associated with prostate enlargement [2]. Numerous epidemiological investigations have revealed a concurrent increase in the prevalence of IR and BPH [3, 4]. However, the potential role of hyperinsulinemia linked to IR in connecting these two conditions has not been thoroughly investigated, which can influence the functional aspects of the prostate gland. Some growth factors associated with metabolism are thought to be significant mediators of the epithelial‐stromal interaction, which is essential for controlling prostate growth [3].

In normal and pathological circumstances, insulin‐like growth factor (IGF) controls development and differentiation [5, 6]. IGF‐1 and its receptor (IGF‐1R) are present in the epithelial cells of prostate tissues [7]. The growth of epithelial cells can be stimulated by insulin and IGF‐1 [8]. IGF‐1 plays a key role in regulating the interaction between the stromal and epithelial cells and contributes to prostate growth [9].

Natural biomolecules have proven potency to regulate blood glucose levels and thus may have possible inhibitory effects on the enlarged prostate gland in conditions of BPH. In this context, exploring the potential role of Capsaicin in BPH is noteworthy. Capsaicin is abundantly obtained from chilli peppers [10] which is pungent flavoured and has many pharmacological effects, including antioxidant [11], anti‐tumour [12, 13], weight‐lowering [14] and analgesic properties [15, 16]. According to the previous studies, Capsaicin has demonstrated improvement in systemic insulin and glucose homeostasis [17, 18] and modulation of intracellular signalling pathways such as the IGF‐1axis [19, 20]. However, it remains unclear whether the protective effects of capsaicin in BPH are mediated through direct modulation of prostatic IGF‐1 signalling or indirectly through restoration of systemic glucose–insulin homeostasis.

This study demonstrates the protective effects of Capsaicin in hyperinsulinemia induced by a high‐fat diet combined with streptozotocin (HFD + STZ) in rats, highlighting its impact on cellular hyperplasia and enhanced cell proliferation by regulating IGF‐1 signalling.

## 2 Materials and Methods

### 2.1 Drugs and Chemicals

Capsaicin was purchased from AOS chemicals, Streptozotocin was purchased from Sigma (Cat. No. S0130), cholesterol, casein from Hi‐Media, lard was purchased from a commercial vendor, and Rat insulin ELISA kit and IHC‐kit were purchased from Elab Science.

### 2.2 Animals and Experimental Design

**Figure 1:**
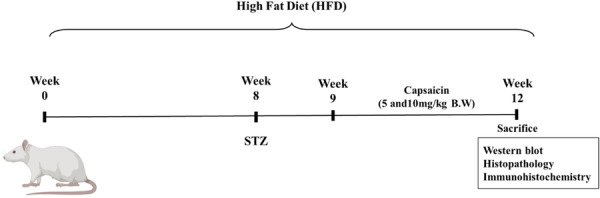
Experimental plan.

Six‐week‐old rats weighing 150–180 g were purchased from Vyas Labs, Hyderabad and acclimatised for 7 days prior to the experimentation. All the animals were maintained at 22°C ± 2°C and 12 h of dark and light. All Experiments were conducted according to CCSEA guidelines (Approval No‐ IAEC/GU‐1287/PRR‐F/2/March 2024). HFD was prepared according to Srinivasan et al, and a normal diet was purchased from Vyas Labs. The composition of the HFD [21] was Powdered normal pellet (365 mg/kg), Cholesterol (10 g), Casein (23 g), protein and mineral mixture (10 mg), Methionine (2 g), Sodium Chloride (2 g). The rats were fed with HFD for 8 weeks, then intraperitoneally injected with 35 mg/kg of STZ dissolved in citrate buffer. After 2 days of injection of STZ, blood glucose levels were measured, and the animals with blood glucose levels > 250 mg/dl were considered as IR and were randomly allocated into different groups. Capsaicin was administered by dissolving in corn oil and given orally once in a day until the day of sacrifice. The animals were further divided into four groups with six animals per group: Control, HFD + STZ group, Capsaicin (5 mg/kg) group, and Capsaicin (10 mg/kg) group. The experimental plan shown in Figure 1.

### 2.3 Glucose Tolerance Test (OGTT)

The animals were given 2 g/kg b.w glucose orally on 2 days before sacrifice. Blood glucose levels were measured at 0, 30, 60, 90, and 120 min using a glucometer. The blood was collected using tail vein method [22]. The trapezoid rule was used to determine the area under the curve (AUC).

### 2.4 Blood and Tissue Sample Collection

On the day of sacrifice, the blood samples were collected at 500 µL/rat by cardiac puncture in mildly anaesthesized rats with Ketamine (80 mg/kg)/xylazine (8 mg/kg) through i.p administration. At the end of the experiment, the rats were euthanised using carbon dioxide asphyxiation and the death of the animals were confirmed by checking respiratory arrest [23]. Blood was centrifuged at 5000 rpm for 5 min in order to separate the serum. The prostate glands were carefully excised. Tissues were immediately fixed in 10% formalin for 24–48 h to preserve morphological integrity for histopathological examination and Immunohistochemistry. The remaining prostate tissue was stored at –80°C for molecular studies.

### 2.5 Biochemical Estimations (Fasting Glucose, Insulin, HOMA‐IR)

Fasting glucose and insulin levels were assessed to determine the development of Insulin resistance in the experimental animals. Before measuring the glucose and insulin levels, the rats were fasted overnight with free access to water. Blood glucose was measured by collecting blood from the tail vein. Insulin levels were measured by using a commercially available ELISA kit specific for rat insulin. The degree of insulin resistance was quantified by Homeostatic Model Assessment for Insulin Resistance (HOMA‐IR). The formula for HOMA‐IR [24].

HOMA‐IR = Glucose (mmol/L) × Insulin (µU/mL)/22.5.

### 2.6 Western Blot

The prostate tissue was lysed by using Radio immunoprecipitation assay (RIPA) buffer supplemented with 1% protease inhibitor. Subsequently, the homogenate was centrifuged at 12,000 g for 15 min at 4°C, and the collected supernatant was used for analysis. Protein quantification was done by Lowry's method. After quantification, samples were mixed with a sample buffer and placed on a heated at 95°C for 5 min for denaturation. For electrophoresis separation, 30 μg of protein sample was loaded, Proteins were separated using Sodium dodecyl‐sulfate polyacrylamide gel electrophoresis (SDS‐PAGE) and then deposited onto the polyvinylidene fluoride (PVDF) membrane [25]. The membrane was blocked for 1 h at room temperature using blocking buffer. The membrane was incubated with primary antibodies [IGF‐1(DF6096, 1:1000), MEK (AF6385 1:1000) and ERK (AF0155 1:2000)] for a whole night at 4°C. After three buffer washes, the membranes were incubated with with HRP‐conjugated secondary antibody anti‐rabbit IgG‐HRP (Cat. No‐31460 diluted at 1:20000), for an hour at room temperature. Later, enhanced chemiluminescence reagent was added for chemiluminescent detection. The blots were washed and visualised using of Vilber Fusion Solo Smart Imager and analysed using Image J software.

### 2.7 Immunohistochemistry

Fixation of tissues was done by formalin, followed by embedding in paraffin. The sections were mounted onto the slide, then tissues were deparaffinised with xylene and rehydrated with graded alcohols. Retrieval of antigen was done by heating in citrate buffer (pH: 6.0) after cooling, and the slides were rinsed with Phosphate buffer [26]. The endogenous peroxidase activity is blocked by incubating with 3% H_2_O_2_ for 10 min. Blocking of non‐specific binding using 5% BSA for 30 min. The sections are incubated with primary antibody IGF‐1(1:200) for 2 h at room temperature and then the slides were washed with PBS. Followed by incubating with horseradish peroxidase (HRP)‐conjugated secondary antibodies, for 1 h in accordance with the manufacturer's instructions and followed by washing with PBS. Diaminobenzidine (DAB) reagent was added and incubated 10 min for signal detection. The slides were counterstained with Mayers haematoxylin (cat. No.48441) and images were captured by Lynx biological Microscope (Model: LM‐52‐1804).

### 2.8 Histopathological Study

The prostate specimens were placed into 10% Formalin for 24 h, The tissues were dehydrated by using a graded alcohol series, then the tissues were embedded in paraffin wax. The tissues were sliced into 4 μm sections and mounted onto a glass slide. These sections were deparaffinized by using xylene, and rehydration was done by a graded series of alcohols [27]. Staining was done by using Haematoxylin and Eosin (H&E). The morphological changes were recorded under a microscope, and images were captured.

### 2.9 Statistical Analysis

Data was expressed as mean ± standard error of mean (Mean± SEM). Among the groups statistical difference was analysed using one‐way ANOVA, followed by Tukey's multiple comparison test. Statistical analyses were conducted with GraphPad Prism version 8.0, and a *p*‐values ≤ 0.05 was regarded as statistically significant.

## 3 Results

### 3.1 Capsaicin Ameliorated Fasting Blood Glucose and Insulin Levels

**Figure 2:**
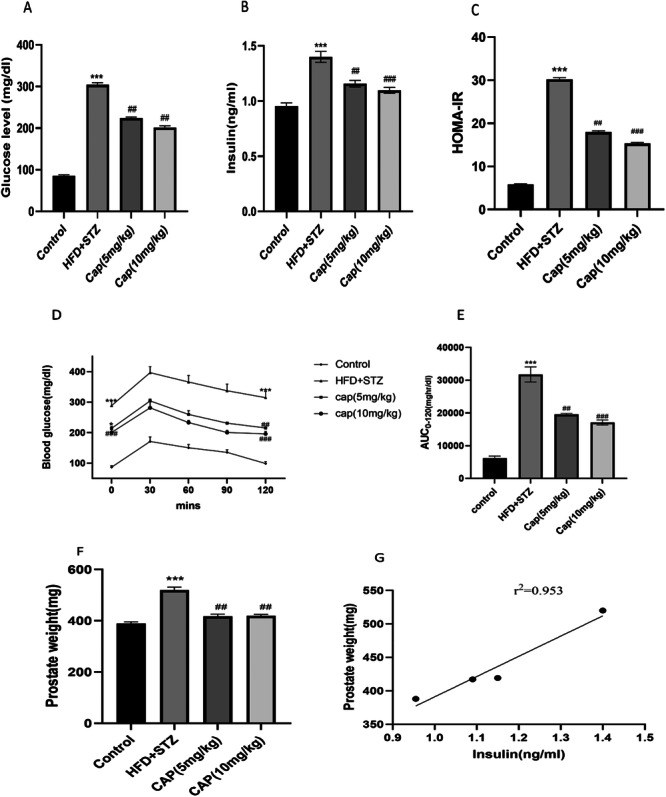
(A) Blood glucose levels, (B) Insulin levels, (C) HOMA‐IR, (D) OGTT, (E) AUC of OGTT, (F) Prostate weights after treatment with Capsaicin, (G) Correlation between Prostate weight and insulin. Values are expressed as the mean ± SEM (*n* = 6) *p < 0.05, **p < 0.01, ***p < 0.001 versus NC, and ^#^ *p* < 0.05, ^##^ *p* < 0.01, ^###^ *p* < 0.001 versus HFD + STZ.

Rats subjected to HFD + STZ treatment developed IR. HFD + STZ rats showed higher insulin and blood glucose (B.G.) levels than the control group (Figure 2A,B). Oral administration of Capsaicin (Cap) to the HFD + STZ group significantly lowered both blood glucose and insulin levels (Figure 2A,B). HOMA‐IR values were high in HFD + STZ (*p* < 0.001 vs control), indicating severe insulin resistance (Figure 2C). HOMA‐IR was significantly reduced in all the treatment groups, reflecting an improvement in insulin sensitivity (Figure 2C). Glucose homeostasis was assessed through an Oral glucose tolerance test OGTT, revealing that Blood glucose levels in the HFD + STZ group were significantly higher, with notable glucose intolerance following exogenous glucose administration compared to control rats. Cap treatment reduced fasting Blood glucose levels compared to the untreated group. Additionally, Cap administration at varying doses resulted in a significant reduction in blood glucose levels over 120 min in comparison to the untreated HFD + STZ rats (Figure 2D). The AUC analysis indicated a reduction in AUC values for the cap‐treated HFD + STZ group compared to the untreated rats (Figure 2E).

### 3.2 Effect of Capsaicin on Insulin Resistance Induced Prostatic Hyperplasia

There was a notable increase in the Prostate weights in the HFD + STZ group in comparison with control rats. Cap treatment significantly inhibited prostate weight when compared to untreated group (Figure 2F). Further, there was high correlation between Prostate weight and insulin level (Figure 2G).

### 3.3 IGF‐1/MEK/ERK Signalling in Prostatic Hyperplasia

**Figure 3:**
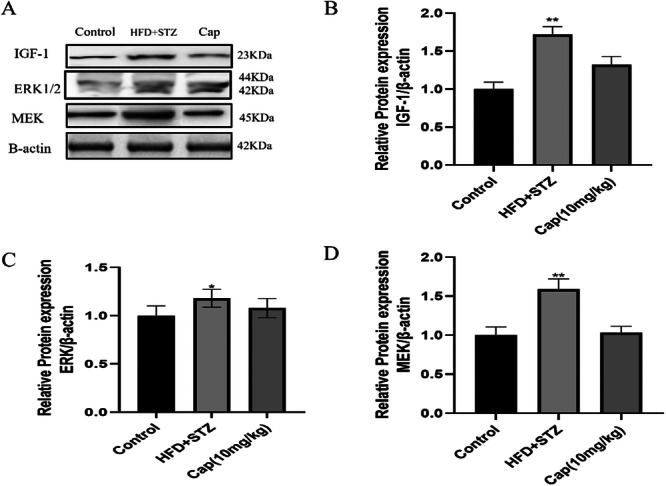
Protein expression of IGF‐1, MEK and ERK. 3. The expression of proteins in the Rat Prostate. (A) Western blot, (B–D) Quantification of IGF‐1, ERK and MEK. Values are expressed as the mean ± SEM (*n* = 3) *p < 0.05, **p < 0.01, ***p < 0.001 versus NC, and ^#^ *p* < 0.05, ^##^ *p* < 0.01, ^###^ *p* < 0.001 versus HFD + STZ, NC‐Normal control, HFD‐high fat diet, STZ‐Streptozocin.

There was a significant increase in IGF‐1 in the HFD + STZ group, indicating upregulation of the IGF‐1 signalling and increased expression of MEK and ERK (Figure 3A–D). To investigate whether Cap regulates IGF‐1 signalling. Treatment with Cap resulted in a significant decrease in the IGF‐1 expression. Cap effectively mitigated prostate growth by modulating the IGF‐1 expression (Figure 3B).

### 3.4 Pathological Changes in Prostatic Tissue

**Figure 4:**
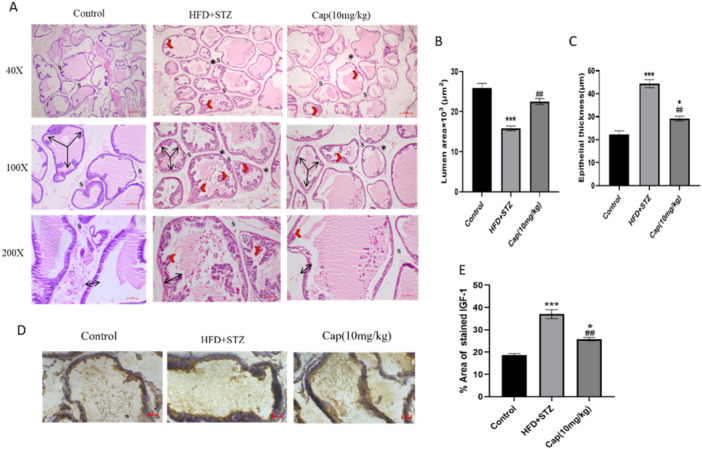
H&E stain & IHC. (A) H&E stain of prostate tissue, the control group did not show infoldings, HFD + STZ group arrow highlights pronounced epithelial hyperplasia, with increased epithelial height and multilayering. There was reduced luminal space and irregular acinar arrangement. In Capsaicin treatment group the arrow points to partially restored acinar structure with wider lumens and reduced epithelial thickening. Acinar infoldings and epithelial thickening (**Red arrow heads**), Stroma (S), inflammatory cells infiltration (**Black asterix**), multi‐direction arrows represent luminal area, Black bidirectional arrows represent the prostatic epithelium height. (B) Graph of luminal space, (C) Representing the Epithelial thickening. (D) Immunohistochemical staining of IGF‐1 in Rat Prostate tissues, Original magnification: 100×; scale bar: 100 μm. Control group showing mild IGF‐1 expression in the epithelial layer, HFD + STZ‐ IGF‐1 expression in both epithelial and stromal regions, and in the Cap treatment group, the intensity of the stain decreased in the epithelial region, and stromal regions show minimal reactivity, similar to the control group. (E) Represents % Area of immunostaining of IGF‐1. Values are expressed as the mean ± SEM (*n* = 3) *p < 0.05, **p < 0.01, ***p < 0.001 versus NC, and ^#^ *p* < 0.05, ^##^ *p* < 0.01, ^###^ *p* < 0.001 versus HFD + STZ, HFD‐high fat diet, STZ‐Streptozocin.

Histopathological analysis showed that the HFD + STZ rats showed a thickened, more infoldings. The control group tissues were found to be normal. Treatment with cap reversed these morphological alterations shown in (Figure 4A). The acinar luminal area showed a significant reduction HFD + STZ group (*p* < 0.001) relative to the control group (Figure 4B). When compared to untreated group, the Cap (10 mg/kg) group revealed significant widening of the acinar lumen in (*p* < 0.01). Statistical analysis of acinar epithelial height (Figure 4C) showed significantly increased epithelial height HFD + STZ relative to the control group. When compared to the untreated group, Cap (10 mg/kg) group had significantly less acinar epithelial height in prostate (*p* < 0.01).

Immunohistochemical analysis revealed a distinct pattern of IGF‐1 expression across experimental groups. The control group showed weak, localised IGF‐1 staining confined to the glandular epithelium (Figure 4D). In contrast, the HFD group exhibited intense IGF‐1 immunoreactivity in both the epithelial and stromal compartments, indicative of metabolic stimulation. Notably, the HFD+ capsaicin‐treated group demonstrated a marked reduction in IGF‐1 staining intensity. This suggests a partial restoration of normal IGF‐1 expression following capsaicin treatment, as shown in (Figure 4E). When compared to the control group, the (HFD + STZ) group expressed significantly higher immunostaining of IGF‐1 (*p* < 0.001, Figure 4E).

## 4 Discussion

Numerous studies have reported that insulin resistance leads to hyperinsulinemia, which in turn contributes to prostatic enlargement [28, 29]. Research has also highlighted a strong association between hyperinsulinemia, insulin resistance, and prostatic enlargement [30]. Beyond its role in regulating plasma glucose levels, insulin exerts growth‐promoting effects. The combination of HFD + STZ is a well‐established model for inducing insulin resistance and hyperinsulinemia. However, a key limitation of this model is that the moderate dose of STZ used may still exert partial effects on pancreatic β‐cell function, which could influence endogenous insulin levels.

In the Present study, IR was confirmed by elevated OGTT and fasting glucose levels in HFD + STZ‐treated rats. Capsaicin treatment at a dose of 10 mg/kg significantly lowered fasting glucose, OGTT, and AUC values. The Prostate weight of HFD + STZ group was higher in comparison to control rats, and this weight gain was accompanied by increased prostatic hyperplasia, further supporting the link between insulin resistance and the progression of prostatic hyperplasia.

Hyperinsulinemia has been linked to elevated levels of IGF‐1, and the IGF signalling pathway is thought to play a role in the connection between IR and prostatic enlargement. Due to structural similarities, insulin can interact with the IGF‐1 receptor, triggering pathways that may influence the growth and proliferation of prostate cells. Research indicates that increased IGF‐1 levels may elevate the risk of BPH [31, 32]. One of the studies a deficiency in IGF‐I has been found to impair prostate development, as demonstrated in studies involving IGF‐I null mice and their wild‐type [33]. Studies suggest that insulin resistance, characterised by hyperglycaemia, hyperinsulinemia, and increased IGF‐1 levels, could contribute to an increased likelihood of prostate hyperplasia [34, 35, 36]. This implies that insulin may drive prostatic growth in insulin‐resistant conditions by activating the IGF pathway, highlighting the importance of a detailed investigation of BPH tissues to understand the underlying mechanisms. Previous report suggests that the IGF‐1 pathway, through activation of MEK and ERK, is involved in the signalling processes and development of BPH in mice [37].

In this study, IR rats exhibited increased prostate weight, along with upregulated IGF‐1 protein expression in the HFD + STZ group. Interestingly, Cap treatment was observed to reduce IGF‐1 expression in the treatment group. The reduced IGF‐1 staining in the treatment group supports capsaicin's therapeutic role in reversing HFD‐induced prostatic alterations.

The research findings indicated an increase in fasting glucose levels, plasma insulin levels, and Prostate weight in the HFD + STZ group in the Rat model. Additionally, IGF‐1 Expression was considerably higher in prostate tissue of hyperplasia cases compared to normal tissue, suggesting a role of the IGF‐1 pathway in the connection between IR and BPH. Capsaicin administration effectively reduced blood glucose and insulin levels in untreated rats and decreased the severity of prostatic hyperplasia. Furthermore, capsaicin also downregulated IGF‐1, MEK and ERK expression, indicating its potential to mitigate prostatic hyperplasia in IR by modulating the IGF‐1 pathway.

The current model is limited to an 8‐week duration study. This short experimental duration may represent an accelerated induction of metabolic stress and prostate changes in contrast to prolonged hormonal and inflammatory milieu contributing to the chronicity of human disease [38, 39]. The doses of capsaicin (5 and 10 mg/kg) were selected based on earlier in vivo studies demonstrating metabolic effects in rats [18] and anti‐proliferative efficacy in mice [40]. Thus, assessment with a longer‐duration study would be prudent to get better insight into the metabolic–prostatic interactions mimicking human insulin resistance–associated BPH. Further, measurement of plasma markers for capsaicin and its metabolites would be more useful to establish optimal therapeutic strategies in BPH associated with insulin resistance.

From a clinical perspective, dietary capsaicin intake—which is typically obtained through eating chillies—may provide protective metabolic benefits requiring additional research into its safety and effectiveness in human trials. From the clinical perspectives, human equivalent doses were calculated using body surface area‐based normalisation [41]. The rat doses correspond to approximately 0.81 and 1.62 mg/kg in humans, which translates to daily intakes of approximately 49–97 mg for a 60 kg adult. Notably, these doses fall within physiologically achievable and potentially tolerable ranges. Overall, the current research offers initial evidence in favour of capsaicin as a possible supplemental dietary or treatment approach for reducing prostatic hyperplasia linked to insulin resistance. To establish the best dosage schedules, more research is needed to understand the pharmacokinetics and bioavailability of capsaicin in humans.

## 5 Conclusion

This study provides compelling evidence that insulin resistance, characterised by hyperglycaemia and hyperinsulinemia, contributes to the development of benign prostatic hyperplasia (BPH), potentially through the activation of the IGF‐1 signalling pathway. HFD + STZ rats exhibited increased glucose levels, insulin levels, prostate weight, and IGF‐1 expression. Capsaicin treatment significantly reduced these parameters, indicating its therapeutic potential. The downregulation of IGF‐1, MEK, and ERK suggests that capsaicin modulates key pathways involved in proliferation. These findings support capsaicin as a promising intervention for BPH associated with insulin resistance.

## 6 Limitations

There are some limitations in the study, as relatively small sample size used for molecular assays such as western blotting and immunohistochemistry, which may reduce the statistical strength of proteomics. Additionally, the 8‐week experimental period is an accelerated model of metabolic dysfunction and prostate enlargement, which might not accurately reflect the long‐term metabolic disturbances linked to the chronic progression of human BPH. Future studies would also include a group treated with a standard BPH medication (e.g., Finasteride) as a positive control to compare the efficacy of capsaicin. To better characterize the long‐term relationship between insulin resistance and prostate pathology, future research with longer durations and larger sample sizes are required.

## Author Contributions

**Manvitha Kotaru:** writing – original draft, methodology, investigation, formal analysis, data curation. **Nihar Ranjan Das:** review and editing, visualisation, supervision.

## Conflicts of Interest

The authors declare no conflicts of interest.

## Data Availability

Data available on request from authors.
